# The Effect of the Cu Interlayer on the Interfacial Microstructure and Mechanical Properties of Al/Fe Bimetal by Compound Casting

**DOI:** 10.3390/ma16155469

**Published:** 2023-08-04

**Authors:** Shiyuan Liu, Hong Xu, Baohong Zhang, Guowei Zhang, Long Bai, Heqian Song, Dan Zhang, Chao Chang, Huan Yu, Chenglong Yang

**Affiliations:** 1 College of Materials Science and Engineering, North University of China, Taiyuan 030051, China; liu_shiyuan1988@163.com (S.L.); zhangbh@nuc.edu.cn (B.Z.); 20030358@nuc.edu.cn (G.Z.); 20210138@nuc.edu.cn (H.S.); 20210114@nuc.edu.cn (D.Z.); lawinzen@163.com (H.Y.); 2 Shanxi Provincial Key Laboratory for Controlled Metal Solidification and Precision Manufacturing, Taiyuan 030051, China; 3 Shanxi Diesel Engine Industry Co., Ltd., Datong 037036, China; fengcun9977@126.com (L.B.); 15235358735@163.com (C.Y.); 4 School of Applied Science, Taiyuan University of Science and Technology, Taiyuan 030024, China; cc@tyust.edu.cn

**Keywords:** Al/Fe bimetal, interface, compound casting, microstructure, bonding strength

## Abstract

Al/Fe bimetals prepared by a compound casting method, combining the excellent properties of both the Al alloy and the ductile cast iron, exhibit great potential for application in achieving engine weight reduction. However, the problem of insufficient interfacial bonding ability because of the difference in thermophysical properties of Al and Fe is particularly prominent. Therefore, in this work, the electrodeposited Cu coating on the surface of the Fe matrix was used as the interlayer of Al/Fe bimetal fabricated by coupling hot-dipping with compound casting to solve the above problem. The effect of Cu interlayer thickness on the interfacial microstructure and shear strength of bimetal was investigated. The experimental results showed that the shear strength up to 77.65 MPa in regard to Al/Fe bimetal with a 5 μm Cu interlayer was obtained. No Cu element was detected at the interface of bimetal regardless of the thickness of the Cu interlayer. The diffusion behavior of the Cu atom at the interface and the influence of the Cu layer at the atomic scale on diffusion reaction and the Al/Fe interface were further revealed by combining first-principle and molecular dynamics calculations. The simulation results revealed that the Cu layer gradually dissolved into an Al alloy at 750 °C, thereby promoting the diffusion reaction of the Al/Fe interface. Meanwhile, the protective role of the Cu layer against oxidation on the surface of the Fe matrix was confirmed. As a result, the interfacial bonding performance was enhanced when the Cu interlayer was introduced.

## 1. Introduction

The bimetal composites formed by the combination of two metals through a specific process technology can give full play to the advantages of both metal materials, thus effectively improving the overall performance [1,2]. Al/Fe bimetal composites, especially, combine the excellent mechanical properties of iron materials and the light weight, high thermal conductivity and corrosion resistance of aluminum alloys and possess a broad prospect in the application of lightweight engine component materials [3,4,5]. The liquid–solid compound casting method has become one of the most widely used processes for preparing Al/Fe bimetal because of its simplicity, low cost and suitability for complex and large structural parts [6]. However, there is a poor wettability between the Al alloy melt and the Fe matrix due to a great difference in physical and chemical properties between Al and Fe, and the contact time between each is short during the casting process, so it is difficult to form an effective fusion zone at the interface of Al/Fe bimetal [7]. These problems seriously affect the interfacial bonding ability of Al/Fe bimetal, thereby restricting the development and application of Al/Fe bimetal. Thus, how to improve the bonding performance of Al/Fe bimetal has important research significance.

A review of the available literature reveals that a coating present on the surface of the iron matrix by surface treatment as the interlayer could improve the wettability of the Al alloy melt on the solid Fe surface [8,9,10]. At present, the surface treatment methods mainly include hot-dipping and electrodeposition, and the coating materials cover Ag, Sn, Au, Ni, Cu, Zn and others [10,11,12,13]. However, the hot-dipping process is influenced by the size of the casting, and the thickness and uniformity of the layer are difficult to control precisely. In contrast, electrodeposition is more suitable for the interlayer preparation of bimetal by compound casting owing to the advantages of simple equipment, convenient and controllable operation as well as low cost [14]. Salimi et al. [15] investigated the effect of aluminizing and Cu electroplating of the steel insert in the fabrication of Al/steel bimetals and found that the r shear strength of bimetals with a Cu coating was higher than that of bimetals with a hot-dipping Al interlayer. Li et al. [12] used the electrodeposited Ni coating as an interlayer to improve the bonding strength of Al/Mg bimetal, and the shear strength reached about 20 MPa. Tavakoli et al. [9] compared the effect of a zinc electroplated coating and hot-dipping interlayer on the microstructure and mechanical properties of Al/steel bimetal and proposed that the maximum strength was related to the electroplated sample with the value of 45.1 MPa. In the previous work [11], the researcher in our team obtained the Al/steel bimetal with interfacial bonding strength up to 115 MPa using an electrodeposited Cr coating as an interlayer. Based on the above, the electrodeposited coating is effective as an interlayer in enhancing the interfacial bonding strength of Al/Fe bimetal.

Although preliminary studies have been conducted on the preparation of electrodeposited Cu coating as an interlayer in bimetals, there is still a lack of research reports on the effect of Cu interlayer thickness on the interfacial microstructure and properties of the bimetal. Therefore, in this work, the mechanism of the Cu interlayer in the bimetal forming process and the law of its thickness on the microstructure and properties will be revealed through a combination of experiments and simulations.

## 2. Materials and Methods

### 2.1. Materials

In this work, ductile cast iron was selected as the solid matrix material, and Al-Si alloy was used as the raw material of the liquid Al alloy melt during hot-dipping and casting. Table 1 and Table 2 give the corresponding chemical compositions, respectively. The electrolyte used for the deposition of the Cu interlayer was from the commercial solution.

### 2.2. Preparation of Al/Fe Bimetal with Cu Coating

Prior to compound casting, the ductile cast iron matrix was machined into tubular substrates with an outer diameter of 41 mm, inner diameter of 36 mm, and height of 65 mm. Then, Cu coating was deposited on the surface of substrates by electro-brush plating equipment. The specific process was conducted in the following steps. The substrate was first subjected to electrical cleaning and then activation treatment, followed by electro-brush plating. Finally, the substrate was cleaned with alcohol and dried in cold air. Among them, it is critical to rinse the substrate with deionized water after finishing each step. The velocity of electro-brush plating was about 400 r/min. As the thickness of Cu coating depends on the deposition time, therefore, the deposition times in this work were 5 min, 10 min and 15 min. Subsequently, the thickness of Cu coating at different times was measured by the thickness tester, and the average of the four measurements was taken as the final value, as shown in Table 3.

During compound casting, the substrates with Cu coating were first preheated to 250 °C for 5 min, followed by hot-dipping in the Al-Si alloy melt at 750 °C for 1 min. After that, the substrates were rapidly placed into a metal mold that was subjected to preheating to 300 °C. Then, the Al-Si alloy melt at 710 °C was poured into the mold around the substrate immediately. When the mold cooled to room temperature, Al/Fe bimetal with Cu interlayer was obtained. Meanwhile, Al/Fe bimetal without Cu interlayer, i.e., the as-casted bimetal, was also prepared for comparative investigation. For convenience, the as-casted bimetal and the bimetal with different thickness Cu interlayer (Cu-5 min, Cu-10 min, Cu-10 min) are represented by Cu-1, Cu-2, Cu-3 and Cu-4, respectively.

### 2.3. Characterizations

The interfacial microstructure of Al/Fe bimetal with and without Cu interlayer was characterized by scanning electron microscopy (SEM) equipped with energy-dispersive X-ray spectroscopy (EDS). Push-out tests were detected using a universal testing machine to evaluate the interfacial bonding property of bimetal under the displacement rate of 1 mm/min; the detail of the test specimens has been reported in the previous literature [11]. The fracture morphology was observed by an optic microscope.

### 2.4. Simulation Method for Interfacial Diffusion Processes

The first-principle calculations were performed by the CASTEP package [16,17], which is based on density functional theory (DFT). The ultrasoft pseudo-potential was used to describe the interactions between ionic core and valence electrons [18]. The PBE (Perdew–Burke–Enzerhof) functional of generalized gradient approximation (GGA) was utilized to treat the exchange correlation [19]. The electronic wave functions were expanded by plane waves up to a kinetic energy cutoff of 400 eV. The convergence criterion for the self-consistent electronic step was set as 1 × 10^−5^ eV/atom, and the structures relaxed until the forces acting on the atoms were less than 0.03 eV/Å. For the pseudo-potential, the electronic configurations were [Al]3s23p1 [Fe]3p63d64s2 and [Cu]3p63d94s2, respectively.

**Figure 1:**
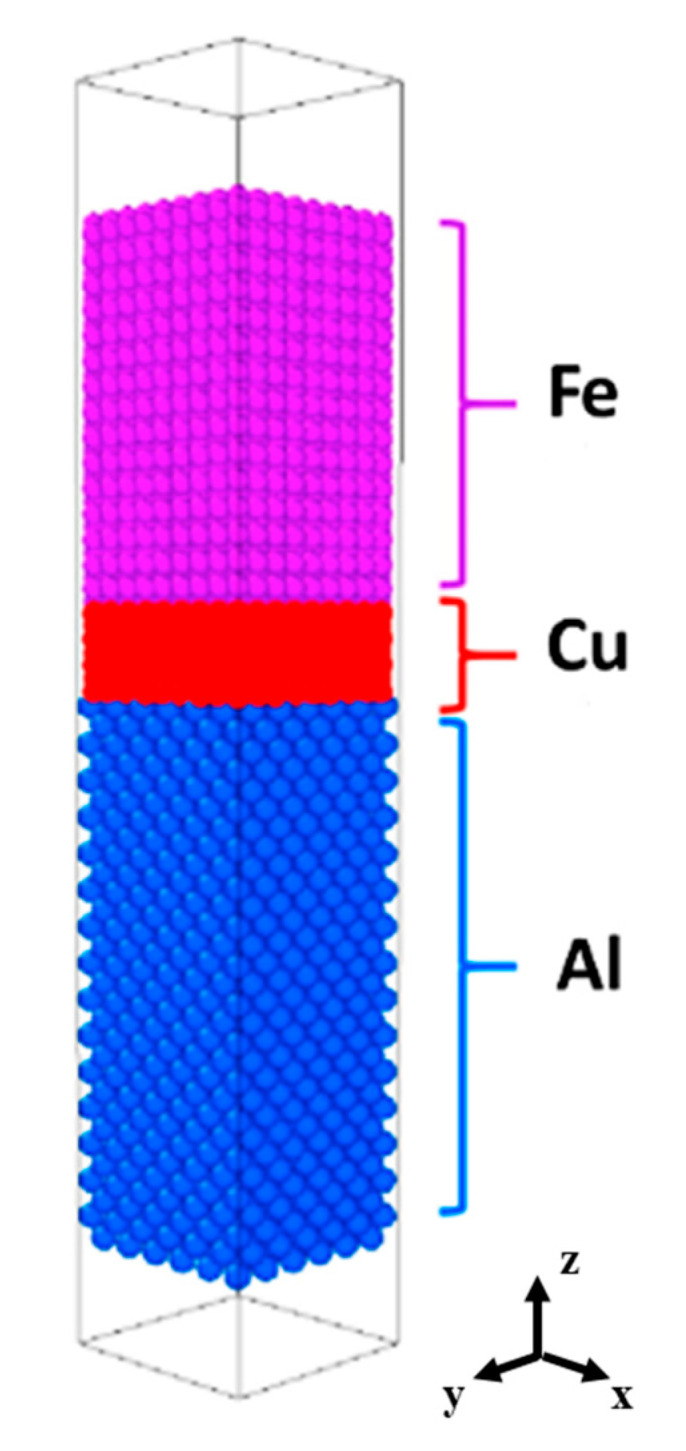
Al/Cu/Fe interdiffusion model.

All of the MD simulations in the present work were conducted with the program Large-scale Atomic/Molecular Massively Parallel Simulator (LAMMPS) developed by Sandia National Labs [20]. The modified embedded atom method (MEAM) potential for Al-Si-Mg-Cu-Fe developed by B. Jellinek et al. [21] was employed to describe the interaction between Al, Fe and Cu atoms. An initial Al/Cu/Fe interface sample, consisting of three independent parts, has been constructed by conjoining at 923–1073 K along the interface normal direction (*z*-direction). The schematic diagram of the Al-Cu-Fe interdiffusion model is illustrated in Figure 1.

## 3. Results and Discussion

### 3.1. Interfacial Microstructure

**Figure 2:**
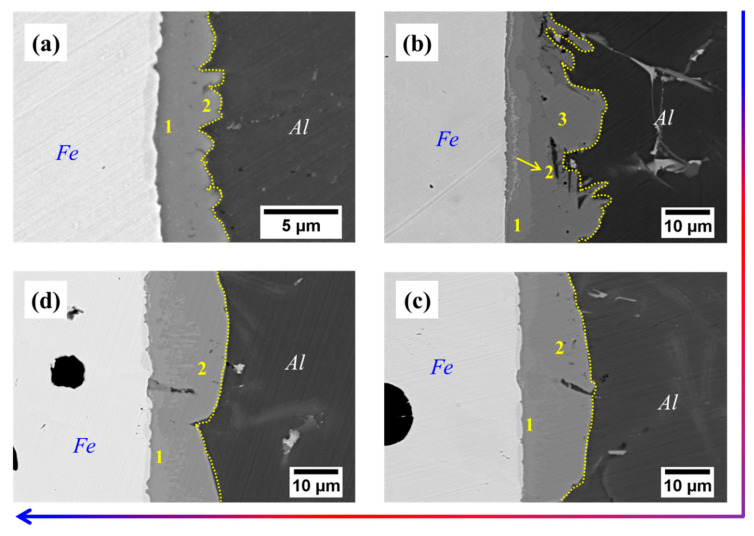
Interfacial morphology of samples: (**a**) Cu-1; (**b**) Cu-2; (**c**) Cu-3; (**d**) Cu-4.

**Figure 3:**
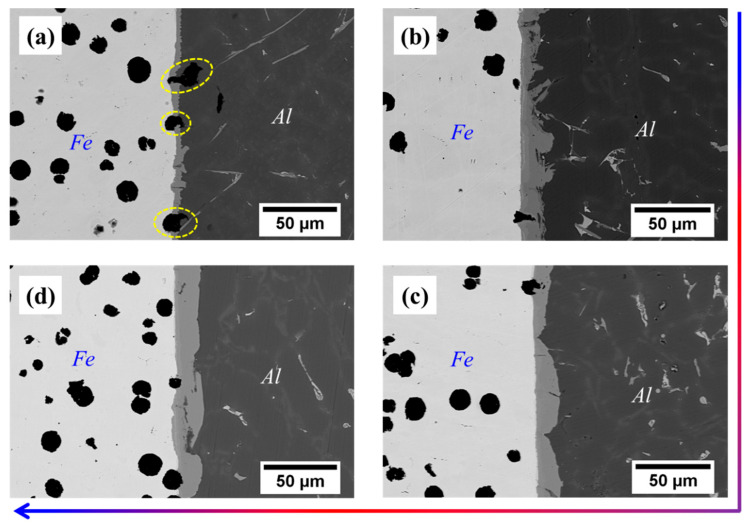
Interfacial morphology at low magnification of bimetals: (**a**) Cu-1; (**b**) Cu-2; (**c**) Cu-3; (**d**) Cu-4.

From Table 3, it can be seen that the thickness of Cu coating tends to increase gradually with the increase of the deposition time, and the thickness of Cu-5 min, Cu-10 min and Cu-15 min is measured as 4.7 μm, 11.5 μm and 26.7 μm, respectively. After compound casting, Al/Fe bimetals with different thicknesses of the Cu interlayer were fabricated, and the interfacial morphology is shown in Figure 2. It is noted that the white area (the left side of each figure) is the Fe matrix, the black area (the right side of each figure) is the casting Al alloy, and the grey area (the middle of each figure) corresponds to the reaction layer. The formation of the reaction layer means that the metallurgical bonding occurs at the interface of all samples. As shown in Figure 2, with the addition of the Cu interlayer and with increasing the thickness of the Cu interlayer, the shape of the reaction layer adjacent to the Al alloy side first increases from a small bump and then becomes smooth. Especially for the Cu-2, the edge of the reaction layer consists of irregular bumps that extend into the Al alloy. Moreover, it is obvious to see from Figure 2b a thin narrow strip existed at the reaction layer that next to the Fe matrix, which is composed of a large number of fine white particles. As shown in Figure 3, at low magnification, however, there are more black graphite particles at the interface of the Cu-1 sample, while this is not the case in the other samples. This reflects that the presence of the Cu interlayer can inhibit the graphite phase on the side of the Fe matrix from participating in the interfacial reaction to a certain extent during the hot-dipping and casting process.

**Figure 4:**
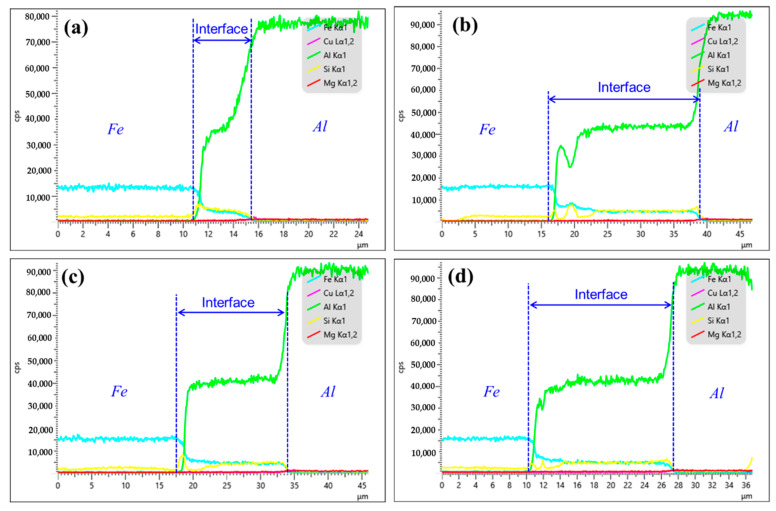
The interfacial elements distribution of bimetal: (**a**) Cu-1; (**b**) Cu-2; (**c**) Cu-3; (**d**) Cu-4.

Figure 4 provides the distribution of the elements along the interface of bimetal. As for Cu-1, the interface is enriched with Fe, Al and Si elements. When the Cu coating was introduced as the interlayer, the interfacial composition of the bimetal was the same as that of the as-casted bimetal, as shown in Figure 4b–d. But it is found that the width of the reaction layer varies greatly. After calculating by Image J software, the thickness for Cu-1, Cu-2, Cu-3 and Cu-4 corresponds to 4.6 μm, 13.0 μm, 16.5 μm and 17.2 μm, respectively. It indicates that the Cu interlayer promotes the metallurgical reaction between the Al melt and the Fe matrix. According to the existing literature, in general, the reaction layer next to the Fe matrix mainly consists of Al and Fe elements, i.e., the Al-Fe phase, and the reaction layer close to the Al alloy is concentrated to Al, Fe and Si elements, namely, Al-Fe-Si phase [22,23]. From Figure 4b, however, the Al-Fe-Si phase appears in the region that is rich in the Al-Fe phase on the near Fe side. Combined with the morphology in Figure 2b and the reports [23,24], it is deduced that the abovementioned region is composed of an Al_2_Fe_3_Si_3_ phase with a white particle shape. In addition, according to Figure 4b–d, the variation curve of Cu element content at the interface approximates a straight line with zero value. It illustrates that, under the conditions studied in this work, all of the Cu interlayers completely dissolve during the hot-dipping or casting process, regardless of their thickness.

In order to identify the effect of the Cu interlayer on the interfacial phase composition of Al/Fe bimetals, the composition of the different regions of the interface was examined, and the results are summarized in Table 4. In terms of the Al-Fe phase diagram [10] and the percentage of atoms in Table 4, it suggests that the reaction layer close to the Fe matrix is mainly composed of Al_3_Fe, Al_5_Fe_2_ and Al_2_Fe_3_Si_3_ phase, while the region adjacent to the Al alloy side is dominated by Al_8_Fe_2_Si phase. This is consistent with what is mentioned in the existing literature [10,22,23,25]. Moreover, the amount of Cu element detected in the reaction layer is almost negligible in regard to Al/Fe bimetals with and without Cu interlayer. This implies that the introduction of the Cu interlayer has no significant effect on the interfacial phase composition of the bimetal. Nevertheless, the increase in the width of the reaction layer, as depicted in Figure 4, reveals a fact that the Cu interlayer promotes the degree of the wettability between the Al alloy melt and the Fe matrix, resulting in a more adequate reaction between Al, Fe and Si at the interface.

### 3.2. Diffusion Behavior of Atoms at the Interface

**Figure 5:**
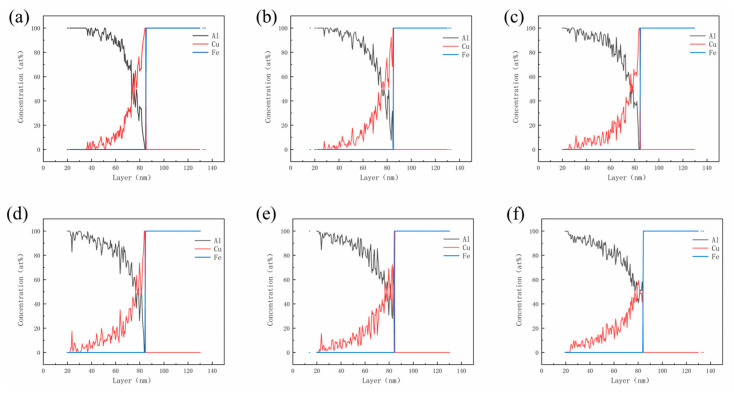
Atomic concentration distribution of Al, Cu, and Fe atoms along the interface at different temperatures: (**a**) 823 K; (**b**)873 K; (**c**) 923 K; (**d**) 973 K; (**e**) 1023 K and (**f**) 1073 K.

The above experimental data reflect that the Cu interlayer is highly susceptible to complete dissolution during the hot-dipping and casting process; therefore, it is necessary to investigate the diffusion behavior of the Cu atom at the interface. Combining first-principles and molecular dynamics calculations, the influence of the Cu layer at the atomic scale on diffusion reaction and the Al/Fe interface is further revealed. This work focuses on the Al(100)/Cu(001)/Fe(001) interface selected based on the mismatch theory. The atomic structure of the Al/Cu/Fe interface is optimized using first-principles calculations, and the surface model of the interface tends to converge with the relaxation of atomic positions for a 7-layered Cu structure. Subsequently, molecular dynamics simulations of interfacial diffusion are performed on the Al/Fe bimetallic model with a 7-layered Cu structure, as depicted in Figure 1. The atomic concentration profiles of Al, Cu and Fe along the z-axis are plotted based on the atomic number density results obtained from LAMMPS simulations with fixed layer thickness. These concentration profiles are shown in Figure 5 at different temperatures (823 K, 873 K, 923 K, 973 K, 1023 K, 1073 K). At 823 K, the Cu layers begin to dissolve, and the diffusion at the Al/Cu/Fe interface mainly takes place between Al and Cu atoms. As the reaction temperature increases, the thickness of the Cu layer gradually decreases, and the diffusion distance of Cu atoms into Al increases. When the temperature reaches 1023 K, the Cu layer at the interface completely disappears, and interactions between Al and Fe atoms start to occur. The main reason for this phenomenon is that the migration rate of Al atoms increases at high temperatures, inducing an increase in vacancy concentration between atoms, further promoting the diffusion of Cu atoms towards the Al side.

**Figure 6:**
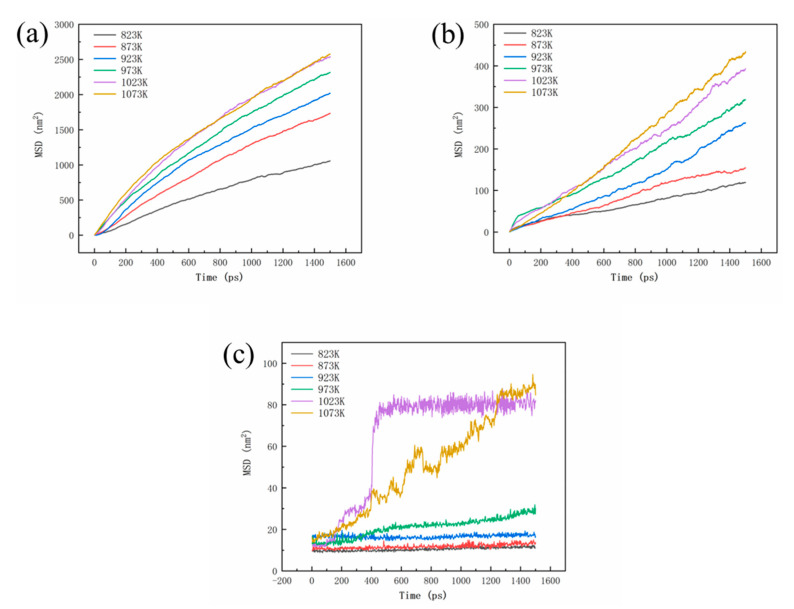
MSD curves for Al (**a**), Cu (**b**), and Fe (**c**) atoms.

Figure 6 shows the mean square displacement (MSD) curves for Al, Cu and Fe atoms. It can be observed that both the MSD curves of Al and Cu atoms increase with increasing temperature, showing a positive correlation with temperature. At temperatures of 1023 K and 1073 K, the MSD values of Al and Cu atoms at the interface are similar, and their increasing trends are also close, indicating a similar diffusion characteristic between Al and Cu atoms. Within the temperature range of 823 K to 973 K, the MSD curve of Fe atoms remains relatively small, and it increases slowly with the diffusion time in the initial stages of diffusion. However, at temperatures above 1023 K, the growth rate of the MSD curve for Fe atom in the initial stages of diffusion significantly increases, followed by a plateau stage. This is mainly due to a complete dissolution of the Cu layer at 1023 K, leading to the interaction between Al and Fe atoms and promoting the tendency of diffusion towards the Al layer. In this process, the diffused atoms have a chemical reaction with the original metal [26]. The simulation results illustrate that the Cu layer gradually dissolves into Al alloy at high temperature, and when the Cu layer completely dissolves, the interface diffusion reactions between Al and Fe atoms occur. A small concentration of any impurity can modify the diffusion rate during crystal growth [27]. This also demonstrates the protective role of the Cu layer against oxidation on the surface of the Fe matrix. These computational simulations are consistent with the experimental observations mentioned above. Thus, it can be concluded that the Cu interlayer will be dissolved completely and promote the interaction between Fe and Al atoms at 750 °C.

### 3.3. Mechanical Property

**Figure 7:**
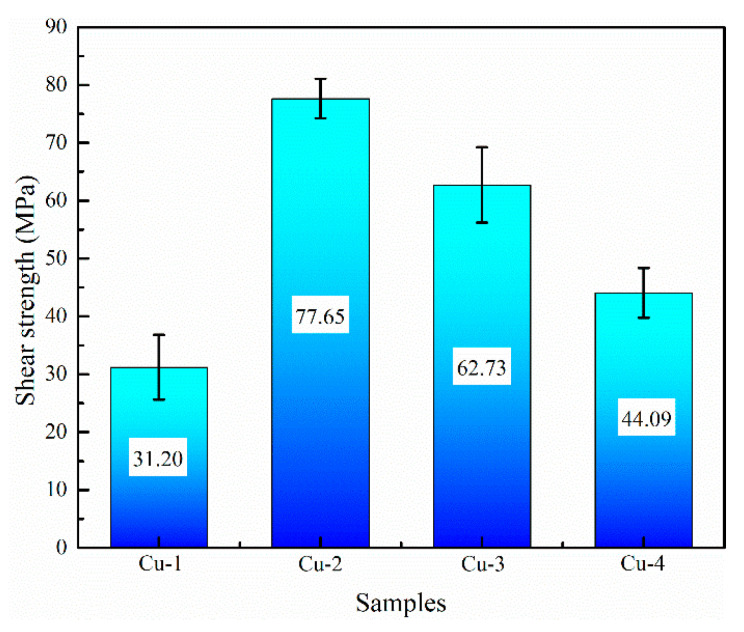
The shear strength of samples.

**Figure 8:**
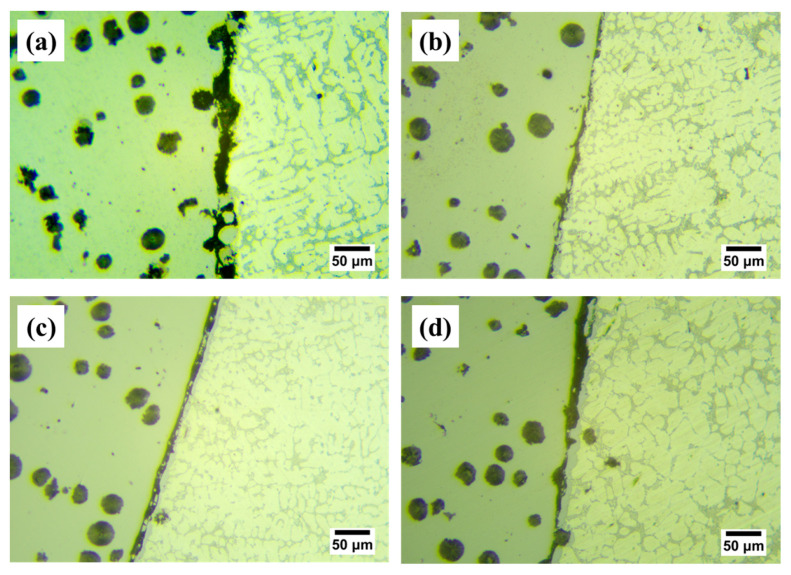
OM images of the interface morphology after shear test for Cu-1 (**a**), Cu-2 (**b**), Cu-3 (**c**) and Cu-4 (**d**).

Figure 7 shows the shear performance test results of the four samples. As for the Cu-1 sample, the shear strength is 31.2 MPa, while the shear strength is further increased when the Cu interlayer is introduced. The shear strength shows a trend of increasing and then decreasing with the increase of Cu interlayer thickness. The maximum shear strength reaches 77.65 MPa for Cu-2, which is about 1.5 times higher than that of the as-casted bimetal. The result fully confirms that the introduction of Cu interlayer can significantly improve the interfacial bonding ability of Al/Fe bimetal. Figure 8 shows the interface morphology after the shear test of all of the samples. As can be seen from Figure 8, all samples fractured at the reaction layer regardless of whether the Cu interlayer was introduced or not. But the difference in morphology can still be observed. There are more black graphite particles at the fracture region with respect to Cu-1. It is thus inferred that these brittle graphite particles are the main reason for the lower shear strength of the as-casted Al/Fe bimetal, as the brittle graphite present at the interface becomes a crack source due to its uncoordinated deformation during the shear process. Compared to Cu-1, the high interfacial bonding strength of Cu-2 is closely related to the following three factors. First, the electrodeposited Cu coating avoids the formation of an oxide film on the surface Fe matrix during preheating. Second, the introduction of Cu interlayer can hinder the contact between the Fe matrix and Al alloy melt to a certain extent, inhibiting the graphite particles in the Fe matrix from participating in the metallurgical reaction at the interface, thereby contributing to the formation of a continuous reaction layer. Third, the hound-like bump structure, as shown in Figure 2b, increases the contact area between the reaction layer and the Al alloy side, thus making it necessary to overcome more potential barriers during the deformation process [12].

## 4. Conclusions

In this work, the electrodeposited Cu coating was used as the interlayer of Al/Fe bimetal prepared by compound casting to improve the interfacial bonding strength. The experimental data revealed that when the thickness was 4.7–26.7 μm, the Cu interlayer was completely dissolved during the fabrication of Al/Fe bimetal. The simulation result also confirmed that the Cu layer gradually dissolved into Al alloy at high temperature, and when the Cu layer completely dissolved, the interface diffusion reaction between Al and Fe atoms occurred. The reaction layer width was increased after introducing the Cu interlayer, but the morphology of the reaction layer near the Al alloy side gradually became smooth from a rough boundary as the thickness of the Cu interlayer increased. The phase composition of the reaction layer was not significantly affected due to the dissolution of the Cu interlayer. Nevertheless, the shear strength of Al/Fe bimetal with a 5 μm Cu interlayer was about 1.5 times higher than that of as-casted bimetal. This was mainly attributed to the barrier of the Cu interlayer to the oxide film on the surface of the Fe matrix, the dissolution of the Cu interlayer contributing to the adequate interfacial reactive layer, as well as the special structure of the reaction layer adjacent to Al alloy side increasing the contact area.

## Figures and Tables

**Table 1:** Chemical composition of ductile cast iron (at.%).

Elements	C	Si	Mn	S	P	Mg	RE	Fe
Content	3.6–3.8	2.5–2.9	<0.6	<0.025	<0.08	0.03–0.05	0.03–0.05	Else

**Table 2:** Chemical composition of Al-Si alloy (at.%).

Elements	Si	Mg	Cu	Mn	Ti	Fe	Sr	Al
Content	6–8	0.25–0.5	1.2–1.8	0.1–0.25	0.1–0.2	≤0.15	0.01–0.06	Else

**Table 3:** Average thickness of Cu coating with different deposition times.

	Position-1	Position-2	Position-3	Position-4	Average
Cu-5 min	4.8 μm	4.9 μm	4.3 μm	4.8 μm	4.7 μm
Cu-10 min	11.3 μm	12.0 μm	11.1 μm	11.6 μm	11.5 μm
Cu-15 min	26.5 μm	26.2 μm	27.2 μm	26.7 μm	26.7 μm

**Table 4:** Chemical composition of different regions in Figure 2 (at.%).

Samples	Regions	Al	Fe	Si	Cu	Possible Phases
Cu-1	Spot 1	73.1	23.4	3.1	0.4	Al_3_Fe + Al_5_Fe_2_
	Spot 2	69.5	18.0	12.1	0.4	Al_8_Fe_2_Si
Cu-2	Spot 1	69.0	28.2	2.7	0.1	Al_5_Fe_2_
	Spot 2	50.3	33.2	16.5	-	Al_2_Fe_3_Si_3_
	Spot 3	71.1	17.8	11.1	-	Al_8_Fe_2_Si
Cu-3	Spot 1	70.7	25.9	3.2	0.2	Al_5_Fe_2_
	Spot 2	70.0	18.8	11.1	0.1	Al_8_Fe_2_Si
Cu-4	Spot 1	69.6	24.6	5.8	-	Al_5_Fe_2_
	Spot 2	70.2	19.5	10.3	-	Al_8_Fe_2_Si
